# Lipidomic analysis of immune activation in equine leptospirosis and *Leptospira*-vaccinated horses

**DOI:** 10.1371/journal.pone.0193424

**Published:** 2018-02-23

**Authors:** Paul L. Wood, Margaret Steinman, Erdal Erol, Craig Carter, Undine Christmann, Ashutosh Verma

**Affiliations:** 1 Metabolomics Unit, College of Veterinary Medicine, Lincoln Memorial University, Harrogate, Tennessee, United States of America; 2 Veterinary Diagnostic Laboratory, University of Kentucky, Lexington, Kentucky, United States of America; 3 Center for Infectious, Zoonotic and Vector-borne Diseases, College of Veterinary Medicine, Lincoln Memorial University, Harrogate, Tennessee, United States of America; Cornell University, UNITED STATES

## Abstract

Currently available diagnostic assays for leptospirosis cannot differentiate vaccine from infection serum antibody. Several leptospiral proteins that are upregulated during infection have been described, but their utility as a diagnostic marker is still unclear. In this study, we undertook a lipidomics approach to determine if there are any differences in the serum lipid profiles of horses naturally infected with pathogenic *Leptospira* spp. and horses vaccinated against a commercially available bacterin. Utilizing a high-resolution mass spectrometry serum lipidomics analytical platform, we demonstrate that cyclic phosphatidic acids, diacylglycerols, and hydroperoxide oxidation products of choline plasmalogens are elevated in the serum of naturally infected as well as vaccinated horses. Other lipids of interest were triacylglycerols that were only elevated in the serum of infected horses and sphingomyelins that were increased only in the serum of vaccinated horses. This is the first report looking at the equine serum lipidome during leptospiral infection and vaccination.

## Introduction

Leptospirosis is a worldwide zoonotic disease that affects horses and many other mammalian species, including man [1]. *Leptospira interrogans* serovar Pomona is commonly associated with clinical leptospirosis in horses in the United States [2, 3] The disease in horses is mainly characterized by spontaneous abortions and recurrent uveitis, with leptospiral abortions occurring late in gestation, typically without any prior clinical signs [4]. Infected mares shed leptospires in the urine for up to 14 weeks and can potentially be a source of infection to other animals. Recurrent uveitis is an important sequela to leptospiral infection and a major cause of blindness in horses [5]. A leptospiral serosurveillance conducted in 2012, reported a prevalence of 45% among horse population in 29 states of the United States and a Canadian province [6].

The microscopic agglutination test (MAT) is the gold standard in serodiagnosis of leptospirosis. The MAT is performed by mixing serial dilutions of patient serum with a battery of live *Leptospira* serovars, and the presence of leptospiral antibodies in the serum is detected by dark-field microscopic examination for agglutination [7]. Among several obvious limitations to the MAT is the test’s inability to distinguish between leptospiral antibodies generated as a result of natural infection from that by vaccination. Vaccinated horses have antibodies to leptospiral bacterin and give positive agglutination reactions in MAT. A test that overcomes the technical limitations of the MAT and distinguishes between infected and vaccinated horses would improve the diagnosis of equine leptospirosis. Recent advances in leptospiral research has resulted in identification of a number of immunogenic leptospiral proteins that are either exclusively expressed or significantly upregulated during infection in horses, but their usefulness in differentiating infected and vaccinated horses is still under investigation [8, 9, 10]. As a result, there currently are no diagnostic tests to differentiate these two immune responses. Alterations in lipid metabolism due to pathogen-induced immune activation have previously been reported [11, 12, 13, 14, 15]. In this study, we asked if differences in host’s responses to live, multiplying *Leptospira* versus killed leptospires, present in the vaccine, are reflected in the serum lipidome of these two groups of horses. To that end, we used a non-targeted lipidomics approach to compare serum lipidome of horses with leptospiral infection and horses vaccinated with a commercially available bacterin.

## Materials and methods

### Serum samples

Fifteen serum samples each from these three groups of horses were used in the study: (1) unvaccinated, naturally infected (Microscopic agglutination test (MAT)-positive) horses, (2) horses vaccinated with Lepto EQ Innovator (Zoetis Inc., Kalamazoo, MI), and (3) unvaccinated, unexposed (MAT-negative) horses (Table 1). Initial screening was performed by MAT, following OIE protocol (http://www.oie.int/fileadmin/Home/eng/Health_standards/tahm/2.01.12_LEPTO.pdf). Naturally infected horses were never vaccinated and had a MAT titer of 1:200 or higher (Table 1). Horses in the vaccinated group did not have a history of prior exposure to *Leptospira* spp. but ruling out any prior exposure is not possible. The control group horses were never vaccinated and had no known history of a prior exposure.

**Table 1:** MAT titers of the serum samples used in the study.

Control sera	MAT titer	Vaccinated sera	MAT titer	Infected sera	MAT titer
UV1	Neg	LV1	1:800 (P, G)	LE1	1:3200 (P)
UV2	Neg	LV2	1:1600 (P); 1:800 (G)	LE2	1:800 (P)
UV3	Neg	LV3	1:3200 (P); 1:100 (G)	LE3	1:400 (P)
UV4	Neg	LV4	1:3200 (P); 1:800 (G)	LE4	1:400 (P)
UV5	Neg	LV5	1:400 (P); 1:100 (G)	LE5	1:800 (P)
UV6	Neg	LV6	1:800 (P); 1:200 (G)	LE6	1:1600 (P)
UV7	Neg	LV7	Neg (P, G)	LE7	1:400 (I)
UV8	Neg	LV8	1:400 (P, G)	LE8	1:200 (I)
UV9	Neg	LV9	1:100 (P, G)	LE9	1:200 (I)
UV10	Neg	LV10	1:200 (G)	LE10	1:12800 (P)
UV11	Neg	LV11	1:100 (G)	LE11	1:3200 (G)
UV12	Neg	LV12	1:800 (P); 1:200 (G)	LE12	1:6400 (P); 1:100 (G)
UV13	Neg	LV13	1:200 (P); 1:100 (G)	LE13	1:25600 (P); 1:400 (G)
UV14	Neg	LV14	1:200 (P)	LE14	1:200 (P); 1:3200 (G)
UV15	Neg	LV15	1:6400 (P); 1:3200 (G)	LE15	1:1600 (P)

MAT, microscopic agglutionation test; P, serovar Pomona; G, serovar Grippotyphosa; I, serovar Icterohaemorrhagiae.

All vaccinated and six of the fifteen samples in the infected group were sent to the UKVDL for MAT titers. Remaining nine samples in the infected group were collected from two different farms in Virginia and Kentucky. Five milliliters of blood was obtained from the jugular vein of horses using a vacutainer needle (20G, 1.5”), a sleeve, and a 10 ml dry blood collection tube (red top). Clotted blood samples were centrifuged at 2,000 x g for 15 minutes. Serum was separated, stored frozen at -20°C, and when required, shipped on dry ice. None of the samples were thawed more than 2 times before the lipidomic analyses were done.

The samples used in this study were left-over aliquots of either clinical diagnostic samples (University of Kentucky Veterinary Diagnostic Laboratory) or blood samples collected in a phlebotomy teaching lab. The phlebotomy lab protocol was approved by the Lincoln Memorial University's Institutional Animal Care and Use Committee.

### Lipid extraction and analysis

For the lipid extraction, 100 μL of serum were vortexed with 1 mL of methanol containing stable isotope internal standards ([^2^H_4_]DHA, [^2^H_4_]hexacosanoic acid, [^2^H_7_]cholesterol sulfate, [^2^H_5_]MAG 18:1, [^13^C_3_]DAG 36:2, [^2^H_31_]PtdE 34:1, [^2^H_54_]PtdE 28:0, [^2^H_31_]PtdC 34:1, [^2^H_54_]PtdC 28:0 and bromocriptine [16–18]. Next 1 mL of water and 2 mL of methyl-tert-butyl ether were added and the tubes were vigorously shaken at room temperature for 30 min. The tubes were next centrifuged at 5,000 xg for 15 min at room temperature and 1 mL of the upper organic extracts was dried by centrifugal vacuum evaporation and dissolved in isopropanol: methanol: chloroform (4:2:1) containing 7 mM ammonium acetate. Constant infusion lipidomics were performed utilizing high-resolution (140,000 at 200 amu) data acquisition, with sub-millimass accuracy on an orbitrap mass spectrometer (Thermo Q Exactive) with successive switching between polarity modes.

In negative ion ESI, the anions of ethanolamine plasmalogens (PlsE), phosphatidylethanolamines (PtdE), lysophosphoethanolamines (LPE), phosphatidylglycerols (PG), phosphatidic acids (PA), lysophosphatidic acids (LPA), cyclic phosphatidic acids (cPA), phosphatidylinositols (PI), ceramides, phosphatidylserines (PS) were quantitated and lipid identities validated by MS/MS.

In positive ion ESI, the cations of choline plasmalogens (PlsC), hydroperoxy PlsCs, phosphatidylcholines (PtdC), lysophosphocholines (LPC), sphingomyelins (SM), monoacylglycerols (MG), and acylcarnitines (ACar), and the ammonium adducts of diacylglycerols (DG), triacylglycerols (TG), and cholesterol esters (CE) were quantitated and lipid identities validated by MS/MS. In the case of hydroperoxy PlsCs, structural identities were validated by the loss of H_2_O_2_ with MS^2^ and generation of a major fragment for choline phosphate.

The cations and anions of bromocriptine were used to monitor for potential mass axis drift. Between injections, the transfer line was washed with successive 500 μL washes of methanol and hexane/ethyl acetate/chloroform (3:2:1) to minimize potential ghost effects.

### Statistical analysis

R values (ratio of endogenous lipid peak area to the peak area of an appropriate internal standard) per 100 μL of serum were calculated. Data are presented as mean ± SEM. Data were analyzed by ANOVA followed by the Dunnets test to compare the vaccinated and infected groups to the controls. Individual data values are available in the Supplementary Information (S1 Table)

## Results

### Cyclic phosphatidic acids (cPA)

**Fig 1:**
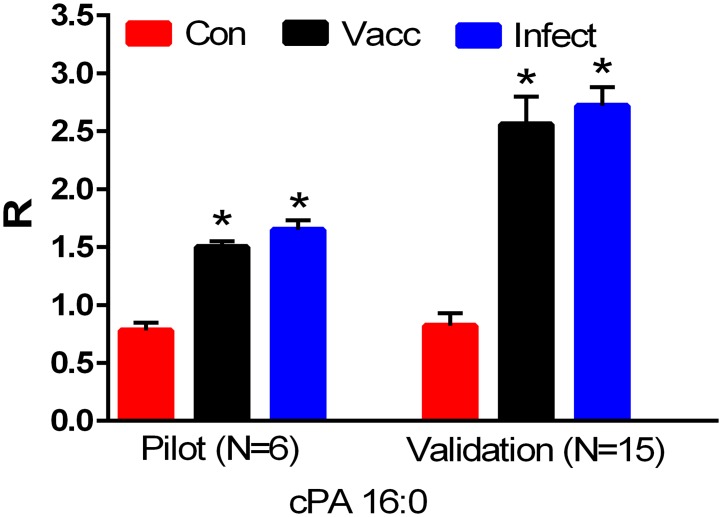
Equine serum levels of cyclic phosphatidic acid 16:0 (cPA) in a pilot study (N = 6 per group) and a validation study (N = 15 per group). Con: controls; Vacc: vaccinated; Infect: infected. *, p < 0.01. R = ratio of the ion intensity for the endogenous cPA to the ion intensity of the stable isotope internal standard.

**Fig 2:**
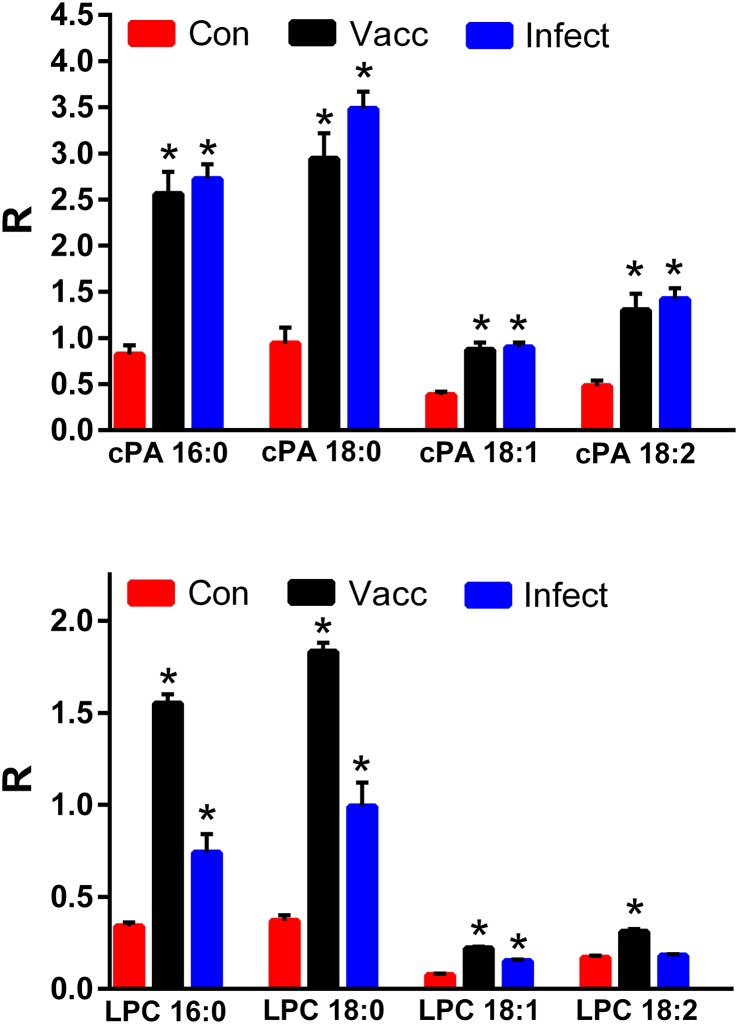
Equine serum levels of cyclic phosphatidic acids (cPA 16:0, cPA 18:0, cPA 18:1, and cPA 18:2) and lysophosphatidylcholines (LPC16:0, LPC 18:0, LPC 18:1, and LPC 18:2) in the validation study (N = 15 per group). Con: controls; Vacc: vaccinated; Infect: infected. *, p < 0.01 vs. controls; #, p < 0.01 vs. the vaccinated cohort. R = ratio of the ion intensity for the endogenous cPA or LPC to the ion intensity of the stable isotope internal standard.

cPA 16:0 (Fig 1) was elevated in the serum of both the vaccinated and infected equine groups. Tandem mass spectrometry validated the structure of cPA 16:0, with the anions for 16:0 and glycerophosphate (-H_2_O) being monitored with 1.2 and 0.98 ppm mass errors, respectively (Table 2). Other cyclic phosphatidic acids also were elevated in the serum of both the vaccinated and infected groups. These included cPA 18:0, cPA 18:1, and cPA 18:2 (Fig 2). The structures were validated by MS^2^ with the product anions for glycerophosphate (-H_2_O) monitored for all 3 cPAs and the respective fatty acid constituents 18:0, 18:1, and 18:2 monitored (Table 2). These data clearly demonstrate that the Th1-type immune responses initiated by leptospirosis [19] or by vaccination [20, 21] result in the generation of cPAs and that these may be important as indicators of immune activation. Of particular interest, horse LV7, while vaccinated, did not demonstrate a MAT titer (Table 1). In contrast cPA changes similar to other vaccinated horses were detected, indicating that this lipid is a more sensitive indicator of immune activation.

**Table 2:** Molecular anions of cyclic phosphatidic acids (cPA) and the associated products monitored with tandem mass spectrometry.

cPA	Calc. Anion	Obs. ppm^1^	Calc [Fatty acid]^-^	Obs ppm^2^	Calc [GP-H_2_O]^-^	Obs ppm^3^
16:0	391.2255	2.4	255.2329	1.2	152.9958	0.98
18:0	419.2568	2.1	283.2637	2.2	152.9958	0.98
18:1	417.2411	2.1	281.2480	2.5	152.9958	1.0
18:2	415.2255	2.0	279.2324	2.1	152.9958	.098

Cal., calculated;

^1^, observed cPA parts per million mass error;

^2^, observed MS^2^ product [fatty acid substituent] parts per million mass error;

^3^, observed MS^2^ product [glycerophosphocholine -H_2_O]^-^ parts per million mass error.

**Fig 3:**
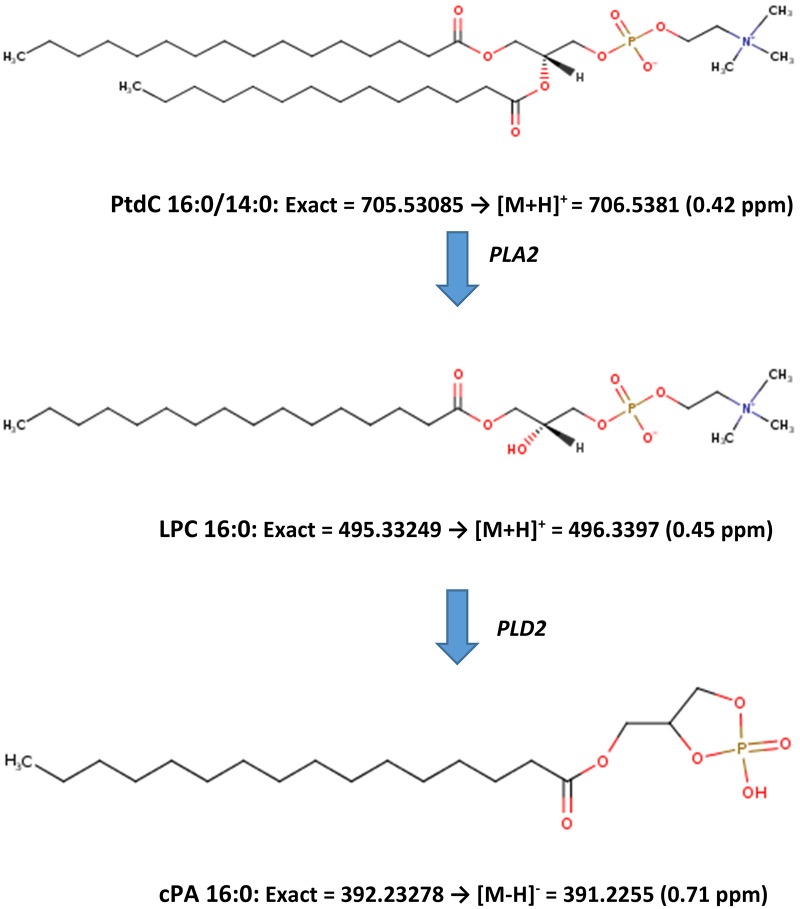
Biosynthetic pathway for cyclic phosphatidic acids (cPA). LPC: lysophosphatidyl-choline; PLA2: phospholipase A2; PLD: phospholipase D; PtdC: phosphatidylcholine. ppm, parts per million mass error.

Cyclic phosphatidic acids are generated by phospholipase D-dependent transphosphatidylation of lysophosphatidylcholines [22], which are generated by phospholipase A2 hydrolysis of phosphatidylcholines [23] (Fig 3). In parallel with augmented cPAs, we monitored increased serum levels of the associated lysophosphatidylcholine (LPC) precursors (Fig 3), albeit, the increases in LPC levels were greater in vaccinated compared to infected horses (p < 0.01).

### Neutral lipids

**Fig 4:**
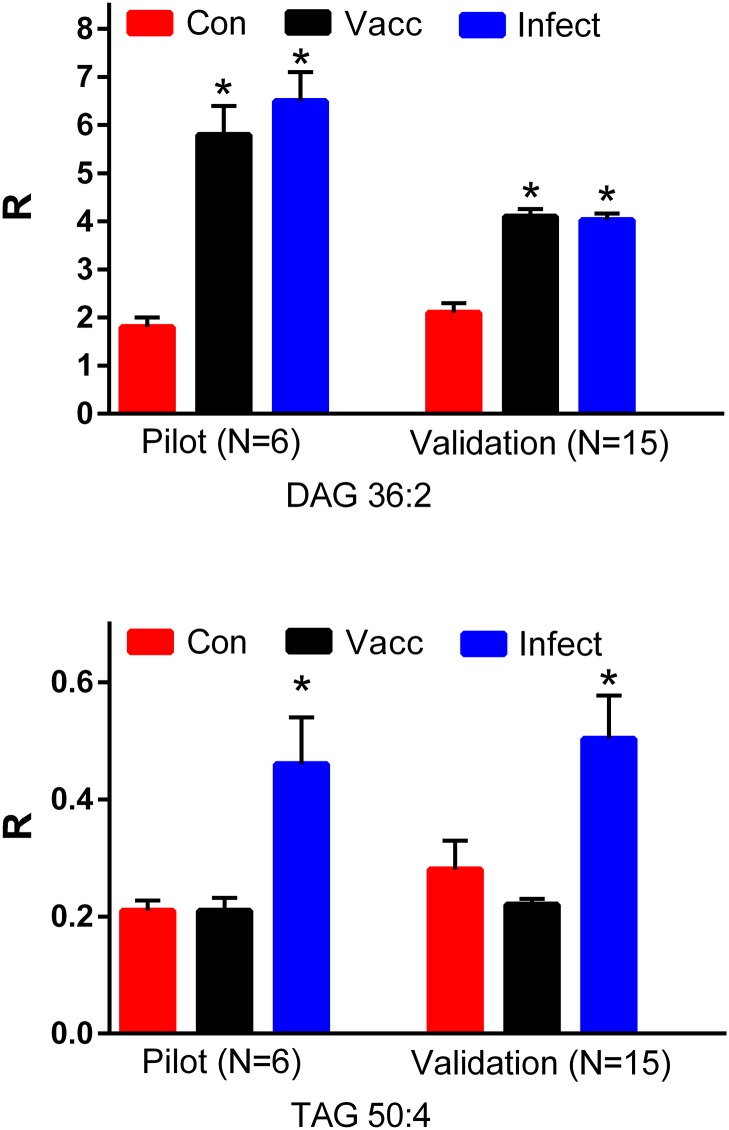
Equine serum levels of diacylglycerol 36:2 (DAG 36:2) and triacylglycerol 50:4 (TAG 50:4) in a pilot study (N = 6 per group) and a validation study (N = 15 per group). Con: controls; Vacc: vaccinated; Infect: infected. *, p < 0.01. R = ratio of the ion intensity for the endogenous TAG to the ion intensity of the stable isotope internal standard.

Diacylglycerols (DAG) also were elevated in the serum of both vaccinated and infected horses. These included DAG 34:1, DAG 34:2, DAG 34:3, DAG 36:1, DAG 36:2 (Fig 4), DAG 36:3, DAG 36:4, and DAG 36:5. Of particular interest, horse LV7, while vaccinated, did not demonstrate a MAT titer (Table 1). In contrast DAG changes similar to other vaccinated horses were detected, indicating that this lipid is a more sensitive indicator of immune activation.

In contrast, triacylglycerols were only increased in the serum of infected horses. These included TAG 48:1, TAG 48:2, TAG 48:3, TAG 50:1, TAG 50:2, TAG 50:3, TAG 50:4 (Fig 4), TAG 50:5, TAG 52:1, TAG 52:2, TAG 52:3, and TAG 52:4.

### Sphingomyelins (SM)

**Fig 5:**
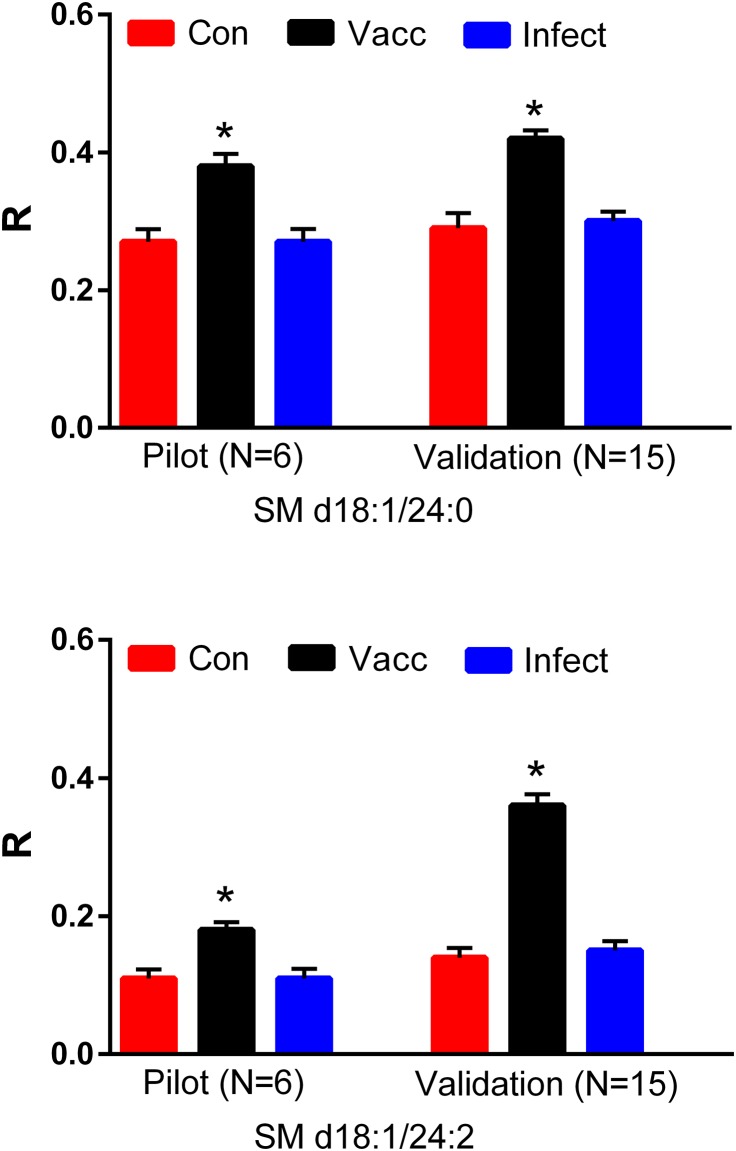
Equine serum levels of sphingomyelins (SM), SM d18:1/24:0 and SM d18:1:24:2 in a pilot study (N = 6 per group) and a validation study (N = 15 per group). Con: controls; Vacc: vaccinated; Infect: infected. *, p < 0.01. R = ratio of the ion intensity for the endogenous SM to the ion intensity of the stable isotope internal standard.

From evaluations of sphingolipids we noted that sphingomyelins were selectively elevated in the serum of vaccinated horses. These included SM d18:1/18:3, SM d18:1/20:0, SM d18:1/22:1, SM d18:1/22:3, SM d18:1/24:0 (Fig 5), SM d18:1 /24:1, SM d18:1/24:2 (Fig 5), and SM d18:1/24:3. In contrast SM changes similar to other vaccinated horses were detected, indicating that this lipid is a more sensitive indicator of immune activation.

While infections generally result in the induction of serine palmitoyltransferase and thereby augmentation of sphingomyelin synthesis [11], this is not universally noted.

### Hydroperoxy glycerophospholipids

**Fig 6:**
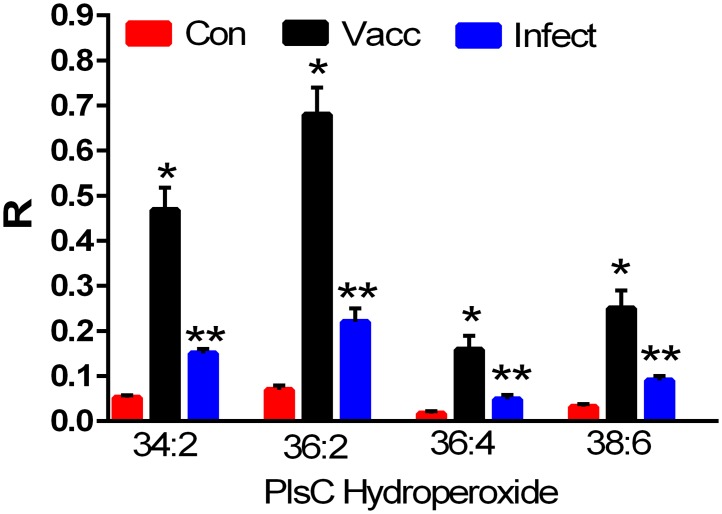
Equine serum levels of choline plasmalogen (PlsC) hydroperoxides (N = 15 per group). Con: controls; Vacc: vaccinated; Infect: infected. *, p < 0.01; **, p < 0.05. R = ratio of the ion intensity for the endogenous hydroperoxide to the ion intensity of the stable isotope internal standard.

The hydroperoxy oxidation products of a number of choline plasmalogens possessing unsaturated fatty acid substituents were detected in infected and vaccinated horses but were greater in the vaccination group (Fig 6). The identities of these oxidation products were validated via tandem mass spectrometry. Using this approach we monitored the loss of both H_2_O and H_2_O_2_ (Table 3) and the generation of choline phosphate (184.0739; < 1 ppm mass error), hallmark features for this class of oxidized lipids [24, 25].

**Table 3:** Molecular anions observed for the hydroperoxy oxidation products of serum choline plasmalogens (PlsC) and their associated MS^2^ products.

Hydroperoxy Lipid	Calc. Anion	Obs. ppm^1^	Calc [M-H_2_O]^-^	Obs ppm^2^	Calc [M-H_2_O_2_]^-^	Obs ppm^3^
PlsC 34:2	774.5643	0.81	756.5537	0.26	740.5588	2.4
PlsC 36:2	802.5956	0.38	784.5850	0.37	768.5901	1.8
PlsC 36:4	798.5643	1.3	780.5537	0.52	764.5588	2.3
PlsC 38:6	822.5643	1.9	804.5537	0.61	788.5588	2.4

Cal., calculated,

^1^, observed hydroperoxy lipid parts per million mass error;

^2^, observed MS^2^ product [M-H_2_O]^-^ parts per million mass error;

^3^, observed MS^2^ product [M-H_2_O_2_]^-^ parts per million mass error.

## Discussion

There is an ever increasing knowledge base regarding the biochemistry of the immune response during infections and inflammatory diseases. A shift in the Th1 and Th2 responses generally results in up-regulation of Th2-type pro-inflammatory cytokines with bacterial infections [26, 27], viral infections [28, 29], and parasitic invasions [30]. In addition local immune responses, such as in the lung [31], brain [32], and the intestine in inflammatory bowel disease (Crohn's Disease and Ulcerative Colitis) [33] elicit alterations in the Th1 and Th2 cytokine responses. Pro-inflammatory cytokines act to induce indoleamine-2,3-dioxygenase-1 thereby acting to deplete tryptophan and generate kynurenine. As a result of this activated pathway, the kyneurenine/tryptophan ratio is often used as a surrogate biomarker of immune activation [34, 35].

Lipid metabolism is also altered by the acute phase reactant response. For example, elevated levels of circulating triglycerides have been observed experimentally with cytokine and lipopolysaccharide injections [11,12, 36], as a result of augmented hepatic lipogenesis. Elevated triglycerides have also consistently been reported with bacterial [11,12,13] and parasitic infections [14, 15]. Altered sphingomyelin metabolism has also been reported, with increases in pneumonia patients [37] and decreases in HIV patients [38].

To further evaluate alterations in the serum lipidome during immune activation we took advantage of the opportunity to compare the serum lipidome of horses with active leptospirosis infection [1, 4, 39] and horses vaccinated with a commercial bacterin [40, 41]. Our results show that serum levels of cyclic phosphatidic acids (cPA), diacylglycerols, and hydroperoxide oxidation products of choline plasmalogens were elevated in both vaccinated and naturally infected horses. Perhaps more importantly, we observed that triacylglycerols were only elevated in the serum of infected horses and sphingomyelins were increased only in the serum of vaccinated horses.

In previous and ongoing studies in our lab we have demonstrated increased levels of cPAs in airway surfactant of horses with asthma [42]. Phospholipase A2 [43, 44] and PLD [45, 46] are both augmented during the early phase of infections suggesting that our cPA biomarkers may be simple biochemical readouts of the induction of these enzymes by immune activation (see Fig 3). Of particular note is that our data is the first to demonstrate that vaccination can activate the same enzyme systems. These data suggest that cPAs may be useful as global biomarkers of immune activation during various infections in horses and possibly other animal species. Considering the breadth of bioactivities of this class of lipids [47], their contributions to immune responses may be diverse, particularly since they modulate nuclear function [48,49]. In this regard, pharmacological studies have shown that cPA analogs potently reverse experimental osteoarthritis [50], block immune-induced demyelination [51], and inhibit the growth of cancer cells [52].

Plasmalogens are essential membrane lipids, particularly in lipid rafts [23]. Alterations in these structural glycerophospholipids, induced by lipid oxidation, may play a role in the host’s immune response, particularly in the development of immunity as evidenced by the dramatic increases in circulating hydroperoxy choline plasmalogens in the *Leptospira*-vaccinated animals. The roles of singlet oxygen-oxidation, free radicals and/or oxygenases in the production of these lipids with vaccination remain to be defined.

In this study we demonstrated that triacylglycerols (TAGs) are elevated only in the serum of naturally infected horses. Previous reports also have consistently demonstrated elevated serum levels of TAGs in both experimental and clinically unresolved immune activation [12–15,36], including human leptospirosis [13]. The mechanism involved in immune-dependent hypertriglyceridemia is thought to involve cytokine activation of triglyceride synthesis in the liver [11]. In contrast, DAGs were elevated in both infected and vaccinated cohorts, suggesting that the synthesis or metabolism of these neutral lipids is altered in both resolved and unresolved immune activation. This is the first report of these changes in DAG levels with immune activation. In this regard, the reports of increased expression of PLD [45, 46] during the early phase of infections suggest that this immune-activated mechanism may be involved in the generation of increased levels of DAGS from glycerophospholipids since PLD metabolizes glycerophospholipids to phosphatidic acids, the direct precursors of DAGs [23].

In our study, sphingomyelins were increased only in the serum of vaccinated horses. Sphingomyelin levels have previously been shown to be elevated in patients with pneumonia [37], and melioidosis [53] while they are unaltered in several bacteremic conditions [53] and decreased in AIDS patients [38]. Our data indicate that the immune response induced by vaccination has a more dramatic effect than leptospiral infection on sphingomyelin synthesis in horses. The role of these lipids in the immune response remains to be more clearly defined.

In summary, our results provide important information about differences in serum lipidome of naturally infected and *Leptospira*-vaccinated horses. Since this study utilized diagnostic and clinical samples, a more controlled, time-matched study is required to further ascertain usefulness of the candidate lipids in differentiating vaccine from infection responses to *Leptospira* spp.

## Supporting information

S1 Table (XLSX)
